# Senescence and Type 2 Diabetic Cardiomyopathy: How Young Can You Die of Old Age?

**DOI:** 10.3389/fphar.2021.716517

**Published:** 2021-10-07

**Authors:** Sian M. Henson, Dunja Aksentijevic

**Affiliations:** ^1^ Centre for Translational Medicine and Therapeutics, London, United Kingdom; ^2^ Centre for Biochemical Pharmacology, London, United Kingdom; ^3^ Centre for Inflammation and Therapeutic Innovation William Harvey Research Institute, Barts and The London School of Medicine and Dentistry, Queen Mary University of London, London, United Kingdom

**Keywords:** type 2 diabetes, immunosenescence, diabetic cardiomyopathy, inflammaging, cardiac metabolism

## Abstract

Inflammation is well understood to be a physiological process of ageing however it also underlies many chronic diseases, including conditions without an obvious pathogenic inflammatory element. Recent findings have unequivocally identified type 2 diabetes (T2D) as a chronic inflammatory disease characterized by inflammation and immune senescence. Immunosenescence is a hallmark of the prolonged low-grade systemic inflammation, in particular associated with metabolic syndrome and can be a cause as well as a consequence of T2D. Diabetes is a risk factor for cardiovascular mortality and remodelling and with particular changes to myocardial structure, function, metabolism and energetics collectively resulting in diabetic cardiomyopathy. Both cardiomyocytes and immune cells undergo metabolic remodelling in T2D and as a result become trapped in a vicious cycle of lost metabolic flexibility, thus losing their key adaptive mechanisms to dynamic changes in O_2_ and nutrient availability. Immunosenescence driven by metabolic stress may be both the cause and key contributing factor to cardiac dysfunction in diabetic cardiomyopathy by inducing metabolic perturbations that can lead to impaired energetics, a strong predictor of cardiac mortality. Here we review our current understanding of the cross-talk between inflammaging and cardiomyocytes in T2D cardiomyopathy. We discuss potential mechanisms of metabolic convergence between cell types which, we hypothesize, might tip the balance between resolution of the inflammation versus adverse cardiac metabolic remodelling in T2D cardiomyopathy. A better understanding of the multiple biological paradigms leading to T2D cardiomyopathy including the immunosenescence associated with inflammaging will provide a powerful target for successful therapeutic interventions.

## Introduction

Type 2 diabetes (T2D) is an enormous global medical and economic burden and its prevalence is on the rise with an ever ageing, increasingly obese population. It is a debilitating, chronic disease affecting almost half a billion people worldwide (World Health Organisation, 2021). Once a “privilege” of high-income Western societies, prevalence of T2D has been rapidly rising in low- and middle-income countries (World Health Organisation, 2021; Miranda et al., 2019). In 2016, an estimated 1.6 million deaths were directly caused by diabetes with an additional 2.2 million deaths attributable to hyperglycaemia in 2012 (World Health Organisation, 2021). Therefore, T2D is an increasing risk to human life span. The current COVID-19 pandemic has added to this chronic metabolic disease burden as the patients with underlying obesity and T2D faced significantly poorer prognosis and clinical outcomes (Barron et al., 2020; Dennis et al., 2021). Therefore, there is a definitive, rapidly increasing necessity for improved understanding of fundamental cellular mechanisms in T2D which can, in turn, help the development of improved treatments and innovative diagnostic techniques. T2D is often presented as a multimorbid disease cluster as it is a major risk factor for the premature onset of multiple age-related conditions such as chronic kidney disease, stroke, impaired wound healing, infection, depression, cognitive decline and inflammation (Kannel and McGee, 1979a; Anderson et al., 2001; Beckman et al., 2002; Brem and Tomic-Canic, 2007). T2D is especially a high-risk factor for cardiovascular mortality and cardiac remodelling, with coronary vessel disease and atherosclerosis being primary reasons for the increased incidence of cardiovascular dysfunction (Wilson, 2001; An and Rodrigues, 2006). Nevertheless, a predisposition to heart failure might also reflect the effects of underlying abnormalities in diastolic function that can be detected in asymptomatic patients with diabetes alone (Fein, 1990; Bertoni et al., 2003; Diamant et al., 2003; Emerging Risk Factors et al., 2010). These observations suggest that progression of T2D leads to specific changes to myocardial structure, function, metabolism and energetics collectively termed diabetic cardiomyopathy (dbCM) (An and Rodrigues, 2006).

T2D is more common in an ageing host and “accelerated ageing” has been proposed as a pathogenic mechanism, including cell ageing leading to a complex phenotype termed senescence (Hughes et al., 2020). Over the past 30 years, cellular senescence has been identified as a possible trigger of general tissue dysfunction and ageing phenotypes (Muñoz-Espín and Serrano, 2014; Dookun et al., 2020). Senescent cell load is low in young individuals but increases with ageing. It is evolutionarily conserved amongst species and is a paradigm of “antagonistic pleiotropy”, an evolutionary hypothesis postulating that traits which are considered beneficial to the organism’s health in early life can exhibit detrimental effects at later stages due to a drop in natural selection pressure (Williams, 1957). Therefore, it can have both detrimental and positive effects dependant on the physiological context. For example, senescence has beneficial effects in the context of wound healing (Demaria et al., 2014), embryonic development (Muñoz-Espín et al., 2013; Storer et al., 2013) and tissue repair; however, when senescent cells accumulate they contribute to tissue dysfunction in the context of ageing and related pathologies (Gorgoulis et al., 2019). Several studies have shown that senescence accumulates in multiple cardiovascular cell populations and is linked with cardiovascular diseases (CVD) including heart failure (HF) (Olivieri et al., 2013; Childs et al., 2017). Furthermore, there are an increasing number of interventional trials in cardiovascular disease and T2D targeting ageing, inflammation and cellular phenotype (Table 1).

**TABLE 1:** Examples of interventional trials in cardiovascular disease and T2D targeting ageing, inflammation and cellular phenotype [table adapted from (Hughes et al., 2020)].

Study name	Study identifier	Study overview	Disease area	Phase	
**Ageing**					
Cardiovascular protective effects of wolfberry in middle-aged and older adults	NCT03535844	An investigation into the protective effects of Wolfberry to change endothelial function and lipidomic profiles in a single-blind 16-weeks study	CVD	N/A
Regulation of endothelial progenitor cells by short-term exercise (EPC-Ex)	NCT01169831	The effect of exercise on endothelial progenitor cell numbers in inactive older adults and endurance athletes via an open interventional study	CVD	N/A
Prevention of cardiovascular stiffening with aging and hypertensive heart disease (LVH)	NCT03476785	An exercise intervention to prevent the age-related stiffening of the left ventricule and vasculature using an open interventional trial	CVD	N/A
n-3 PUFA for vascular cognitive aging	NCT01953705	An investigation into the effects of omega 3 PUFA to support small blood vessels in the brain and brain health in adults >75 years of age at a risk of cognitive decline *via* an interventional study	CVD	2
Impact of ageing on adipose, muscle and systemic inflammation	NCT02777138	Observational study of immune cells and inflammatory markers in adipose tissue between younger and older males with equivalent lifestyles	CVD, T2D	N/A
Cell signaling and resistance to oxidative stress: effects of aging and exercise	NCT03419988	An investigation of the effects of exercise on the expression of nuclear erythroid-2-p45-related factor-2 (Nrf2) in peripheral blood mononuclear cells in healthy younger (18–28 years) and older (>60 years) adults using an open interventional study	CVD, T2D	N/A
Resistance exercise and low-intensity physical activity breaks in sedentary time to improve muscle and cardiometabolic health (REALPA)	NCT03771417	The impact of resistance exercise with low-intensity physical activity on skeletal muscle and cardiometabolic health in adults aged between 65 and 80 years using an interventional study	CVD, T2D	N/A
Dietary reduction of AGEs to prevent cognitive decline in elderly diabetics	NCT02739971	An assessment of feasibility to reduce dietary AGEs and cognitive decline using an intervention pilot study	T2D	N/A
**Inflammation**					
AZD1656 in diabetic patients hospitalised with suspected or confirmed COVID-19 (ARCADIA)	NCT04516759	Assessment of the safety and efficacy of a glucokinase activator (AZD1656) in patients with either Type 1 or Type 2 diabetes, hospitalised with COVID-19. A randomised double-blind, placebo-controlled clinical trial	T2D	2
Effect of IL-1β inhibition on inflammation and cardiovascular risk	NCT02272946	Investigate into the effects of inhibiting IL-1β using canakinumab to limit vascular inflammation in HIV-infected individuals *via* an interventional study	CVD	N/A
ASSessing the effect of Anti-IL-6 treatment in myocardial infarction: the ASSAIL-MI trial (ASSAIL-MI)	NCT03004703	Assessing the impact of a single dose of an anti-IL-6 antibody (tocilizumab) to treat myocardial damage following myocardial infarction, an interventional study	CVD	2
Effects of SGLT-2 inhibition on myocardial fibrosis and inflammation as assessed by cardiac MRI in patients with DM2	NCT03782259	Inhibition of the glucose transporter, SGLT-2, using dapagliflozin and its impact on cardiovascular health and inflammation in patients with T2D, an interventional study	CVD, T2D	4
**Cellular processes**					
Early iNO for oxidative stress, vascular tone and inflammation in babies with hypoxic respiratory failure	NCT01891500	An investigation of inhaled nitric oxide in hypoxic newborns with respiratory failure to reduce biomarkers of oxidative injury *via* an interventional study	CVD	4
Impacts of mitochondrial-targeted antioxidant on peripheral artery disease patients	NCT03506633	The impact of targeted mitochondrial antioxidant (MitoQ) usage on vascular endothelial function in patients with peripheral vascular disease using an interventional study	CVD	N/A
Effects of saxagliptin on adipose tissue inflammation in humans	NCT02285985	An investigation of saxagliptin on adipose tissue inflammation in obese volunteers, an interventional study	T2D	4

In T2D, comorbidities including obesity, hypertension and atherosclerosis all have the ability to increase the number of senescent cells (Minamino and Komuro, 2007; Westhoff et al., 2008; Minamino et al., 2009; Tchkonia et al., 2010). However, the relationship between T2D and myocardial senescence may be both complex and complementary. The microenvironment of systemic metabolic stress in T2D could be permissive to the development and accumulation of senescent cells. On the other hand, senescent cells may contribute to the cardiac parenchyma dysfunction and comorbidities observed in T2D. Overall, it is likely that these complex interactions might lead to a malicious positive feedback in which systemic metabolic dysfunction in the early stages of T2D leads to immune cell senescence that in turn contributes to the worsening of cardiac function and tissue metabolism, which further increases the formation of senescent cells while decreasing their removal (Palmer et al., 2019).

The aim of this review is to spark discussion and help generate hypotheses that may link senescence to cardiometabolic complications in T2D. We hypothesize that clearing senescent pro-inflammatory immune cells or targeting the SASP (Senescence-Associated Secreting Phenotype) may present opportunities for the development of revolutionary therapies for diabetic cardiomyopathy and its complications, leading to advances in its treatment and prevention. Furthermore, we review our current understanding of the metabolic remodelling of both heart tissue and senescent immune cells in T2D, and we discuss potentially fundamental mechanisms by which these metabolic responses influence and intersect each other to ultimately determine the prognosis of the myocardial inflammation.

## Pathophysiology of Type 2 Diabetes

**FIGURE 1:**
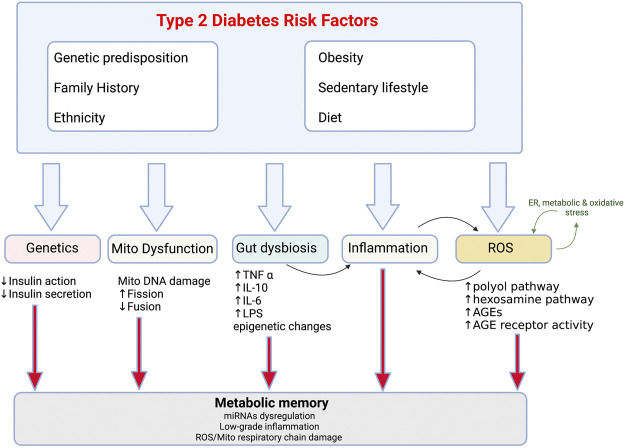
Risk factors and pathophysiological changes in type 2 diabetes Combination of the modifiable (obesity, sedentary lifestyle, diet) and non-modifiable (genetics, ethnicity, family history) risk factors and resultant pathological changes which drive reduction in insulin sensitivity in T2D. Mito-mitochondria, AGE-advanced glycated end products, ROS- reactive oxygen species.

In terms of its clinical manifestation, T2D is heterogenous disease. However, there are two primary pathological mechanisms responsible for the development of T2D: defective insulin production by pancreatic β-cells and insulin resistance (IR) which arises due to the impaired ability of insulin-sensitive tissues to respond to insulin. Multiple organs are involved in the aetiology of type 2 diabetes: pancreas (β-cells, α-cells), liver, skeletal muscle, kidneys, brain, small intestine, and adipose tissue (Defronzo, 2009). Emerging data also suggests an important pathophysiological role of inflammation, adipokine dysregulation, abnormal gut microbiota and immune dysregulation in the development of T2D (Figure 1) (Schwartz et al., 2016). Traditionally, loss of β-cells by cell death has been considered the key cause of β-cell dysfunction in T2D. However, multiple studies have identified β-cell damage caused by lipotoxicity and glucotoxicity-driven endoplasmic reticulum (ER) stress rather than cell loss to be the main cause of impaired insulin secretion (Galicia-Garcia et al., 2020).

IR refers to a decreased response of insulin-sensitive cells to insulin and an impaired response to circulating insulin by blood glucose levels (Czech, 2017). IR can be defined by three broad categories: reduced insulin secretion by β-cells; insulin antagonists in the plasma and impaired insulin tissue response in target tissues (Pearson et al., 2016). The action of insulin is also affected by the interplay of additional molecules such as IGF-1 and growth hormone. IR contributes to increased hepatic glucose production and reduced glucose uptake by muscles, liver and adipose tissue. A diminished insulin action in these target tissues often precedes the development of systemic IR. T2D patients are commonly characterized by obesity [body-mass index (BMI) ≥30 kg/m^2^] and higher body fat percentage especially in the abdominal region.

Obesity is the strongest risk factor for the development of T2D and accompanies metabolic perturbations resulting in IR (Carey et al., 1997; Sinha et al., 2002). It promotes IR through multiple inflammatory mechanisms including adipokine dysregulation and increased release of fatty acids (FA) into circulation. Even if both processes relating to disrupted glucose homeostasis take place early in the pathogenesis of T2D, β-cell dysfunction is considered a more severe cause of T2D than IR. However, concomitant IR and β-cell dysfunction amplify hyperglycaemia leading to worsening of T2D (Cerf, 2013). In addition to the disruption in insulin production, there are a series of pathological conditions which perpetuate T2D including gut dysbiosis, metabolic memory and mitochondrial dysfunction (Figure 1) (reviewed in (Galicia-Garcia et al., 2020)). Furthermore, development of T2D is also affected by the genetics and the environment (Figure 1). Genome-wide association studies have identified the polygenic nature of T2D, as well as identified some of the common glycaemic genetic variants for T2D. These account for 10% of total trait variance highlighting their importance (Grarup et al., 2014). In addition, ethnic origin may lead to different phenotypes that increase predisposition to risk factors such as hypertension, IR and dyslipidaemia (Wong et al., 2016). Genetic factors are exacerbated by the exposure to an environment characterized by obesity and physical inactivity including a high-calorie, Western diet.

## Diabetic Cardiomyopathy

**FIGURE 2:**
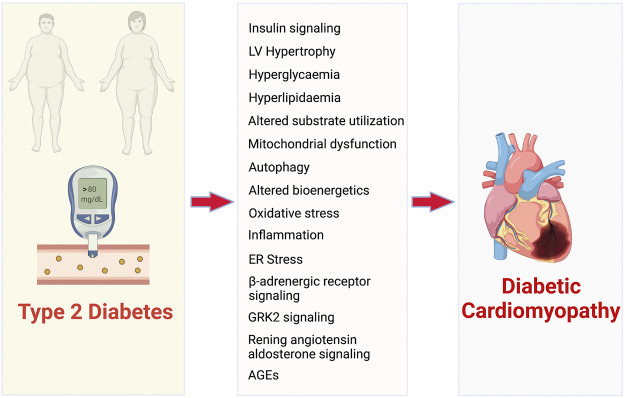
Pathophysiological mechanisms that contribute to the development of diabetic cardiomyopathy and associated heart failure. AGEs- advanced glycated end products.

Accelerated heart failure (HF) is a common manifestation of cardiovascular disease in T2D (Shah et al., 2015). Approximately £3 billion of the £10 billion total cost of diabetes to the UK National Health Service (NHS) is associated with the cardiovascular complications of diabetes, and in the next 20 years this figure is projected to double (Hex et al., 2012). The Framingham Heart Study concluded that T2D independently increases the HF risk up to 2-fold in men and 5-fold in women compared to matched controls (Kannel et al., 1974; Kannel and McGee, 1979b). Furthermore, adjusting for other risk factors including age, hypertension, hypercholesterolemia, and coronary artery disease does not eliminate increased HF incidence. 40 years ago, the term “diabetic cardiomyopathy” (dbCM) was defined to initially define ventricular dysfunction in the absence of hypertension and coronary artery disease in T2D patients (Kenny and Abel, 2019). However, over the years it has been identified that the progression of T2D leads to specific changes to myocardial structure, function, metabolism and energetics, collectively termed diabetic cardiomyopathy (dbCM) (An and Rodrigues, 2006). Thus, the use of the term “diabetic cardiomyopathy” has been extended to describe the increased susceptibility of the T2D heart to dysfunction. The mechanisms by which T2D leads to dbCM have been extensively reviewed elsewhere (Kenny and Abel, 2019; Marelli-Berg and Aksentijevic, 2019; Tan et al., 2020). However, the principal pathways that contribute to the development of dbCM and diabetes-associated HF have been summarised in Figure 2.

## Type 2 Diabetic Environment Promotes Senescence

**FIGURE 3:**
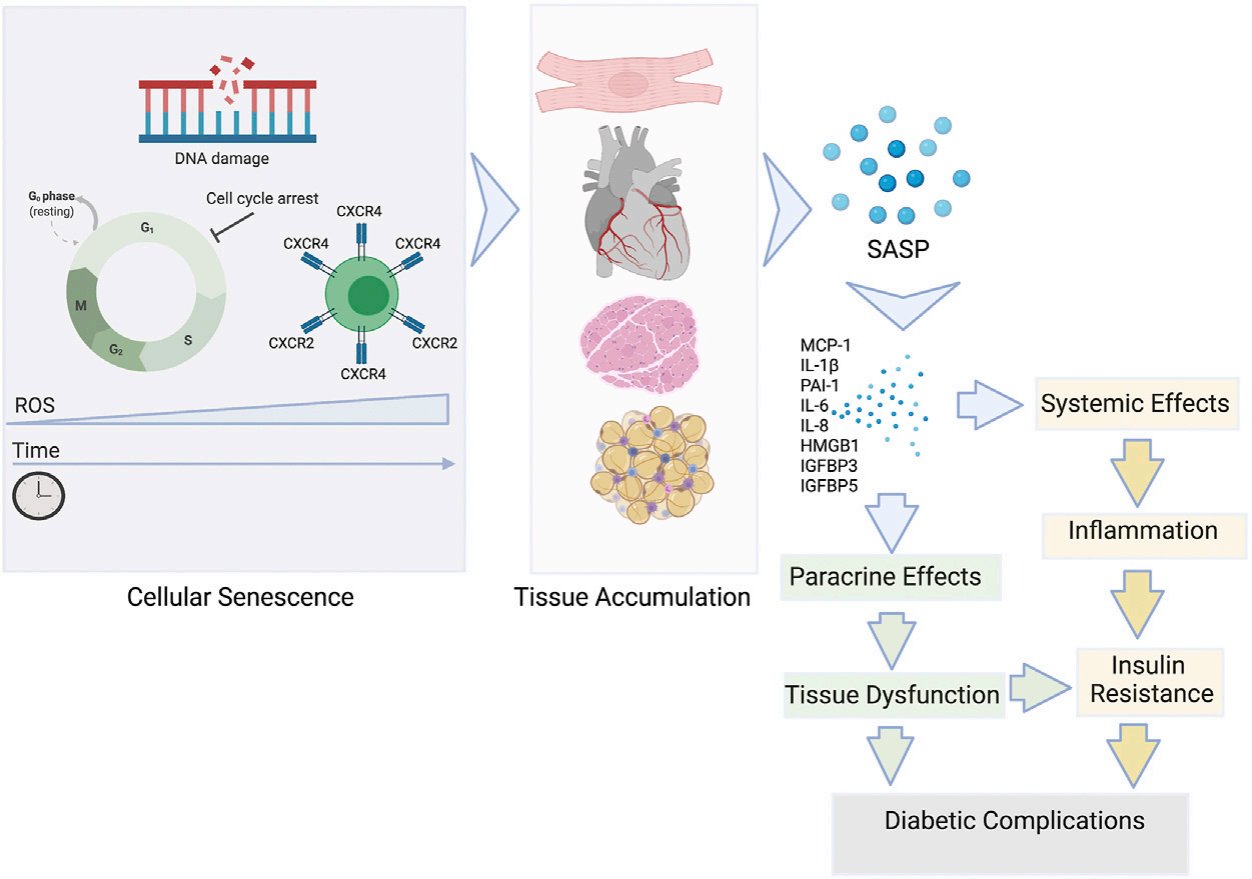
The role of senescent cells in the development of insulin resistance and T2D complications. Senescent cells accumulate in tissues during ageing and the development of pathophysiological states. The SASP is a diverse group of proinflammatory mediators: cytokines, chemokines, growth factors released by the senescent cells systemically and locally. The SASP can affect the function of surrounding cells via paracrine signalling contributing to tissue dysfunction and ultimately damage that can cause diabetic complications. SASP factors can also enter the circulation and add to the chronic inflammatory state commonly associated with the insulin resistance development.

Cellular senescence is thought to have originated as the anti-tumour mechanism in response to oncogenic activation and by definition includes irreversible growth arrest in response to cellular stresses such as DNA damage, oxidative stress and telomere erosion (Figure 3). Despite the inability to divide, senescent cells maintain high metabolic activity supporting the secretion of growth factors, proinflammatory cytokines, chemokines, angiogenetic factors, and matrix metalloproteinases collectively defined as the Senescence-Associated Secreting Phenotype or SASP (Coppé et al., 2008; Kuilman and Peeper, 2009).

Production of the SASP empowers even a small number of senescent cells present in any organ (ie <20%) to exert significant, widespread systemic effects (Campisi and d'Adda di Fagagna, 2007). Senescent cells also have the ability to initiate a damaging positive feedback mechanism by promoting the spread of senescence to neighbouring cells (Zhu et al., 2014; Tchkonia et al., 2013; Nelson et al., 2012). This potentially includes obesity-related senescence promoting chronic, low-grade sterile inflammation. Driven by the primary hallmarks of ageing, there is low-grade inflammation in older individuals which is similar to the systemic inflammation seen in chronic disease, a phenomenon termed “inflammaging” (Franceschi and Campisi, 2014). This may be the crucial link between underlying obesity and chronic inflammation leading to cardiac metabolic dysfunction in T2D (Figure 3). SASP mediators have also been shown to reduce peripheral insulin sensitivity, enhance chemoattraction and activate pro‐inflammatory macrophages, that further contribute to IR (Palmer et al., 2019). Analogous to the other age-related chronic diseases, T2D may be caused in part by a convergence of the basic ageing mechanisms that underlie age-related tissue dysfunction, including macromolecular damage, chronic sterile inflammation, progenitor cell dysfunction, and cellular senescence (López-Otín et al., 2013). However, the full impact on the cardiac function and metabolism are yet to be fully understood.

## Pancreatic Dysfunction

Pancreatic dysfunction and the resultant impairment in insulin response and hyperglycaemia are the hallmarks of T2D. Several studies have shown that the gene expression in β cells changes with ageing including increased expression of the genes related to cellular senescence, such as *Cdkn2a* and *Cdkn2b* (Helman et al., 2016). Deletion of senescent β cells, *via* senolysis in a pre-clinical diabetic model was found to preserve insulin secretory capacity, providing a link between cellular senescence and severe dysregulation of insulin secretion (Thompson et al., 2019). Furthermore, high-fat diet experimental models have confirmed pancreatic β cell senescence as a key contributor to T2D (Sone and Kagawa, 2005). In transgenic models of T2D, the cell cycle inhibitor p27 (a marker of senescence) increases in pancreatic β cells, and p27 deletion increased insulin secretion and islet mass through increased proliferation (Uchida et al., 2005). In transgenic mice lacking p53-dependent apoptosis, the number of senescent cells rapidly increases and is associated with dysfunction of pancreatic β cells, causing an overt diabetic phenotype (Tavana et al., 2010). This represents an accelerated model of age-related T2D and is consistent with the hypothesis that cellular senescence could result in decreased insulin synthesis and release. Therefore, eliminating senescent cells by senolytic drug agents may possibly prevent b cell dysfunction (Zhu et al., 2015).

## Systemic Metabolic Stress

Hyperglycaemia is a hallmark of T2D and has been shown to drive premature senescence in endothelial cells, renal mesangial cells, adipose-derived stem cells and fibroblasts (Yokoi et al., 2006; Blazer et al., 2002; Ksiazek et al., 2008; Cramer et al., 2010). Whilst the mechanism of hyperglycaemia-induced senescence remains to be understood, potential mechanisms include mitochondrial dysfunction and increased reactive oxygen species (ROS) damage (Ksiazek et al., 2008). High glucose can also enhance the formation of advanced glycation end products (AGEs) (Palmer et al., 2019). Increased AGE signalling through their receptors (RAGE), of which the SASP factor HMGB1 is also the agonistic ligand (Figure 3), has been shown to cause premature senescence in renal tubular cells (Liu et al., 2014; Palmer et al., 2019).

In addition to hyperglycaemia, systemic dyslipidaemia and the resultant lipotoxicity play an important role in the pathophysiology of diabetic cardiomyopathy. Increased intracellular ceramide concentration has been linked to promotion of senescence due to alterations in fatty acid metabolism in response to environmental stress *via* p53 and p38 mediated pathways (Ford, 2010). Synthesis of ceramides is increased in T2D causing systemic tissue damage including apoptosis of pancreatic β cells (Galadari et al., 2013). In fibroblasts and endothelial cells, ceramide exposure increases the expression of senescence markers thus driving the process of cellular senescence (Ford, 2010). Moreover, inhibition of *de novo* ceramide synthesis in ob/ob transgenic mice has been shown to reduce the SASP factors PAI-1 and MCP-1 as well as improve insulin and glucose sensitivity (Yang et al., 2009). Furthermore, conversion of the T2D biomarker 1-deoxysphinganine to its ceramide metabolite 1-deoxy-dihydroceramide, decreases insulin secretion in Ins-1 β cells and primary islets and triggers p21-dependent cellular senescence in Ins-1 cells (Zuellig et al., 2014).

## IGF-1/Growth Hormone

Whilst the relationship between growth hormone, insulin resistance and T2D is complex and still not fully explored, there have been a series of studies suggesting this complex axis plays an important role in triggering senescence. Alterations in the secretion and tissue response to growth hormones in T2D have been implicated in promoting senescence. Components of this pathway such as growth hormone, IGF1 and IGF5 binding protein (IGFBP5) have all been shown to trigger senescence (Kim et al., 2007; Stout et al., 2014; Tran et al., 2014). In addition, IGFBP3, has been implicated in the SASP response, playing a role in the paracrine propagation of senescence to surrounding cells and tissues (Elzi et al., 2012). Indeed, a comprehensive proteomic database of soluble proteins and exosomal cargo SASP factors originating from multiple senescence inducers and cell types has been published (Basisty et al., 2020). A core SASP exists of mediators that are secreted irrespective of the senescent stimuli or cell type investigated, which included IGFBPs. Chronic exposure to IGF-1 in T2D due to hyperinsulinaemia and altered IGFBP levels and can lead to p53-mediated premature senescence (Palmer et al., 2015). Consistent with the role of IGF-1 in senescence, the activity of Akt which is a major downstream target of the insulin/IGF-1 signalling pathway, is increased in senescent cells. Furthermore, *in vitro* endothelial cell experiments have shown that Akt inhibition both delays senescence and increases replicative cellular lifespan (Palmer et al., 2015). IGFBP members IGFBP5 and IGFB3, were shown to increase during endothelial and fibroblast senescence. IGFBP5 may induce cellular senescence on its own *via* a p53-dependent, p16-independent pathways (Palmer et al., 2015) and the levels of IGFBP3 can be modulated by the SASP component PAI-1 (Palmer et al., 2015). IGFBP3 was shown to trigger insulin resistance independently of IGF binding, supported by decreased GLUT4 translocation to the plasma membrane as well as reduced Akt phosphorylation in response to insulin (Palmer et al., 2015). In addition, overexpression of IGFBP3 led to decreased glucose tolerance, insulin resistance, and hyperglycemia (Palmer et al., 2015).

## Cardiomyocyte Senescence

Defining senescence in classic terms in cardiomyocytes is a challenge as they are terminally differentiated cells, necessitating multiple methodologies such as senescence-associated-β-galactosidase (SA-β-gal) staining, quantification of p53, p21 and p16 together with an assessment of the SASP. It should be noted that cellular senescence is a separate process to apoptosis having distinct biological pathways (Childs et al., 2014). Soon after birth, cardiomyocytes undergo cell cycle arrest due to the activation of the DNA damage response introduced through exposure to higher concentration of oxygen in the postnatal environment (Shimizu and Minamino, 2019). Therefore, cell cycle arrest alone cannot be used to define senescence. Instead, the definition of cardiomyocyte senescence includes DNA damage response, ER stress, mitochondria dysfunction, contractile dysfunction, hypertrophic growth, SASP and β-galactosidase expression (Ock et al., 2016; Hernandez-Segura et al., 2018; Tang et al., 2020). Cardiomyocyte senescence is regulated by the intracellular signalling pathways such as the metabolic sensors/regulators and the extracellular microenvironment, including the paracrine effects of the non-myocytes such as endothelial cells, fibroblasts and immune cells. DNA damage is both an important driver and a hallmark of cardiomyocyte senescence, with telomere shortening being the most common DNA damage feature of senescent cells (Shay and Wright, 2019). Evidence from both animal and human studies has shown that post-mitotic cardiomyocyte senescence is mediated by length-independent telomere damage (Ball and Levine, 2005; Anderson et al., 2019). Administration of cytotoxic agents such as doxorubicin were shown to upregulate p16^Ink4a^, p21 and SA-β-gal level (Shirakawa et al., 2016). For example, in the human failing heart, hypertrophic cardiomyopathy (HCM) HfrEF and ischemic cardiomyopathy cardiomyocyte telomeres were shorter compared to healthy controls (Shakeri et al., 2018). DNA damage is also closely linked to alterations in cardiac mitochondrial function as in senescent cardiomyocytes, mitochondrial ROS production and accumulation induces DNA damage and repair response (Tang et al., 2020). A recent study has offered interesting and novel mechanistic insight into how senescence can accumulate in a predominately post-mitotic cardiomyocyte population (Dookun et al., 2020).

It has been shown that accumulation of persistent telomere-associated foci of DNA damage (TAF) is a major driver of cardiomyocyte senescence during ageing. Cardiomyocyte TAF were induced by oxidative stress, as a result of age acquired mitochondrial dysfunction, and occured independently of telomere length and cell cycle activity. Furthermore, DNA damage-induced PARP1 activation contributes to the cardiac contractile dysfunction *via* NAD metabolite depletion (Zhang et al., 2019). Regardless of the stressors triggering the response, DNA damage at telomere regions leads to the activation of a persistent DNA damage response (DDR), due to the inability of telomere regions to undergo non-homologous end joining (Fumagalli et al., 2012; Dookun et al., 2020). This permanent DDR results in the activation of either one or both p53/p21^Cip^ or p16^Ink4a^/retinoblastoma protein cyclin-dependent inhibitor pathways (Dookun et al., 2020). Regardless of the source of damage, senescent cardiomyocytes are characterized by SASP production. This includes CCN family member 1 (CCN1), interleukins (IL1α, IL1β, and IL6), tumour necrosis factor-alpha (TNFα), and monocyte chemoattractant protein-1 (MCP1), endothelin 3 (Edn3), tumour growth factor-beta (TGFβ), and growth and differentiation factor 15 (GDF15) (Dookun et al., 2020). These SASP factors are crucial regulators of non-myocytes within the local microenvironment and contribute to cardiac remodelling and dysfunction in senescent states such as T2D.

One of the key features of the senescent murine and human myocardium is the hypertrophic cardiomyocyte enlargement and increased pro-hypertrophic gene expression (Cui et al., 2018). Furthermore, activation of the inhibitory components of cell cycle regulators have been shown to ameliorate cardiac hypertrophy (Maejima et al., 2008).

## Cellular Senescence and Cardiac Metabolic Dysfunction in Type 2 Diabetes

Senescent cell burden increases in ageing and obesity and may play a role in causing or exacerbating myocardial dysfunction in T2D. There have been a number of studies over the years detailing extensive changes in cardiac metabolism during senescence and T2D, however the metabolic nexus between these comorbidities have not been identified. Decades of cardiac ageing research have helped to understand the alterations in cardiac metabolism associated with the senescence. Both pathological states are characterized by remodelling at the cellular level in the form of left ventricular hypertrophy, development of contractile dysfunction and maladaptive changes in cardiac metabolic profile. It has been shown that senolysis in obese mice led to improved cardiac diastolic function, with important translational implications for T2D cardiomyopathy patients, in whom heart failure with preserved ejection fraction is common (Palmer et al., 2019). Furthermore, as an overarching comorbidity there is a presence of inflammation both in terms of inflammageing of the immune system cells as well as a chronic low-grade inflammation of cardiac parenchyma. However, despite the known link between senescence, inflammation and T2D, the exact relationship between these morbidities has not been established.

The adult heart has an enormous ATP demand in order to support its continuous contractile work as well as in order to deal with the dynamic workload alterations. Continuous supply of ATP for the healthy adult heart is provided by mitochondrial oxidative phosphorylation (90% of ATP synthesis). In cardiac mitochondria, fatty acids and carbohydrates are the main substrates for ATP synthesis *via* oxidative phosphorylation (OXPHOS). Under physiological conditions fatty acid oxidation (FAO) accounts for approximately 70%, whilst the remaining contribution is from oxidation of carbohydrates *via* pyruvate and lactate. Substrate usage by the heart is flexible, as the substrate utilisation ratio is rapidly adjusted in order to maintain a continuous ATP supply (reviewed in (Lopaschuk et al., 2021)). Severe metabolic alterations characterise the diabetic heart, with changes in substrate utilization, alterations in mitochondrial organisation and function, together with enhanced ROS production all being observed and which collectively lead to energetic deficit (reviewed in (Marelli-Berg and Aksentijevic, 2019)).

Due to underlying insulin resistance, myocardial glucose utilization is compromised, accompanied by increase in FA supply. As a result, cardiac ATP synthesis switches to the dependence on FA utilization. High FA uptake and metabolism not only augment accumulation of FA intermediates and triglycerides but also increase oxygen utilization and generation of reactive oxygen species (ROS) causing cardiomyocyte damage (reviewed in (Marelli-Berg and Aksentijevic, 2019)) However, how these metabolic alterations contribute to the senescence of cardiomyocytes in dbCM or whether cardiomyocyte senescence in this scenario, is the *primum movens* of cardiac cells metabolic dysfunction remains unknown. Intermittent hyperglycaemia can induce stress senescence *via* p21 and p16 but sustained hyperglycaemia was found to activated telomerase and shorten telomere length, indicative of replicative senescence (Maeda et al., 2015). There have however been suggestions that altered mitochondrial metabolism may be the pivotal contributor to myocardial senescence thus forming the basis for the increased sensitivity of the T2D heart to stress and functional decline (Lesnefsky et al., 2016). The age-associated senescent cardiomyocyte is characterized by metabolic inflexibility, with a decreased FAO and enhanced dependence on glucose metabolism *via* enhanced PDK4 content (Hyyti et al., 2010; Chiao et al., 2016). Senescence impairs mitochondrial OXPHOS, including decreased activity of mitochondrial complexes III and IV, which account for the decrease in ATP synthesis (Lesnefsky et al., 2016). Furthermore, ageing decreases mitochondrial content, altered fission, fusion, autophagy among the myofibrils resulting in ≈50% decrease in mitochondrial function, impacting all metabolic substrates (Cramer et al., 2010). The presence of defective mitochondria leads to enhanced production of oxidants leading to oxidative injury and the triggering of oxidant signalling for cell death. Thus, hypothetically triggering of senescence by systemic metabolic stress as well as pro-inflammatory cell accumulation during the development of diabetic cardiomyopathy may exacerbate any underlying cardiac metabolic derangement due to altered insulin sensitivity.

ROS-mediated mitochondrial DNA damage and mutations are thought to be a major cause of reduced mitochondrial biogenesis in senescent and diabetic hearts. Mitochondrial polymerase mutation increases oxidative stress in middle-aged mice resulting in cardiomyopathic changes (Tchkonia et al., 2010; Dookun et al., 2020). Threshold for ROS-induced ROS-release is reduced in the aged cardiomyocytes making them more sensitive to mitochondrial permeability transition pore (mPTP) induction. mPTP opening results in mitochondrial swelling leading to impaired ATP provision, apoptosis and oxidative stress. Mitochondria isolated from variety of aged and senescent tissue have suggested that aging affects the opening of mPTP (Panel et al., 2018). Mechanistically, mPTP opening is prevented in mitochondria from young and healthy animals by the high membrane potential (∆Ψ), tightly regulated matrix Ca concentration and ROS scavenging [reviewed in (Panel et al., 2018)]. With ageing there is a progressive loss of mitochondrial cristae due to disassembly of ATP synthase dimers, increase in Ca and production of ROS as well as decline in ∆Ψ. Impaired Ca regulation leads to matrix Ca elevation which is the principal trigger for mPTP opening. Mitochondrial respiratory chain as well as being the main source is also a target of ROS damage. ROS targets multiple sites in mitochondria including respiratory chain complexes leading to enhanced ROS generation and lowering ∆Ψ. Furthermore, enhanced ROS production causes cardiolipin peroxidation which sensitizes mPTP to Ca overload (Panel et al., 2018). The theory of increased mPTP opening in senescent rat heart is also supported by failure of pharmacological mPTP inhibitors to produce effects under baseline or stress conditions. Furthermore, in aged mitochondria cyclosporin A (CsA) is unable to inhibit carboxyatractyloside‐induced permeability transition and GSK‐3β inhibition of SB‐216763 in ischemic injury is abrogated (García et al., 2009; Zhu et al., 2011; Li et al., 2013). Collectively, these data are consistent with increased susceptibility to apoptosis and suggest that mPTP is activated in senescent states (Chabi et al., 2008).

Mitochondrial-specific overexpression of catalase in mice leads to the rescue of premature ageing and senescent cardiac phenotype. This observation supports the premise that mitochondrial ROS production and DNA damage are contributors of a vicious circle of ROS-induced ROS- release, resulting in cardiac dysfunction. Further validation of the role of mitochondria in cardiac senescence was derived from the work in a p66shc transgenic mouse.

p66shc is a mitochondrial redox enzyme that forms ROS using electrons leading to H_2_O_2_ production (Soto-Gamez et al., 2019).

The p66shc mutation decreased oxidative damage in mice thus increasing the lifespan and decreasing cardiac dysfunction (Dookun et al., 2020). Therefore, along with diabetes, senescence-induced oxidative stress could exacerbate the vicious cycle of mitochondrial deterioration.

## Inflammatory Signalling and Metabolic Derangement in Diabetic Cardiomyopathy

Numerous cellular mechanisms may be involved in the development of myocardial inflammation in T2D. Mechanisms are mainly directed towards the activation of the NF-κB pathway. This mechanism is prevalent in diabetic vasculature and myocardium contributing to damage by upregulation of cytokines (TGF-β1, IL-1β, IL-6, IL-18, TNF-α), chemokines and adhesion molecules (Baker et al., 2011; Gordon et al., 2011; Shah and Brownlee, 2016; Marelli-Berg and Aksentijevic, 2019). Furthermore, cardiomyocyte-specific overexpression of IκB-α protein, suppressor of the canonical NF-κB signalling pathway, prevented streptozotocin-induced diabetic cardiomyopathy by inhibiting the renin-angiotensin system (Thomas et al., 2014). Moreover, pharmacological inhibition of NF-κB in T2D has been shown to reduce mitochondrial abnormalities and reduce myocardial oxidative stress (Mariappan et al., 2010). Chronic low-grade myocardial inflammation driven by metabolic stress may also cause cardiac dysfunction by triggering metabolic perturbations that impair energetics.

For example, cardiac glucose utilization was impaired by IL-6 administration *via* SOCS3-dependent inhibition of IRS-1 (Frati et al., 2017). In contrast, IL-6 gene mutation reduced inflammation and rescued glucose metabolism defects triggered by high-fat diet, accompanied by SOCS3 inhibition and IRS-1 reactivation (Ko et al., 2009). Moreover, myocardial PGC-1α expression, a principal regulator of mitochondrial biogenesis and function (Shah and Brownlee, 2016), has been inhibited by a chronic inflammation *via* NF-κB activation (Palomer et al., 2009). This may be caused by binding and sequestering of PGC-1α by p65, thereby resulting in downregulation and inhibition (Alvarez-Guardia et al., 2010). Systemic metabolic derangements in T2D has also been shown to activate NF-κB: hyperglycaemia-triggered activation of the PKC pathway *via* DAG, increased hexosamine pathway flux, increased AGEs, and increased polyol pathway flux (Du et al., 2003). Common feature of all outlined pathways is increased ROS production and activated nuclear poly-(ADP-ribose)-polymerase (PARP) (Du et al., 2003). PARP over-activation in hyperglycaemia drives NAD^+^ synthesis *via* the salvage pathway which consumes ATP (Szabó, 2006). Furthermore, this process results in ribosylation and inactivation of glyceraldehyde-3-phosphate dehydrogenase (GAPDH), resulting in increased intermediates of glycolysis thus activating the proinflammatory transcription factor (Du et al., 2003). Several studies link inflammation and T2D and are associated with increase in CRP and IL-6 (Pradhan et al., 2001). Additional mechanisms for increased inflammation in dbCM include activation of NADPH oxidase, endoplasmic reticulum (ER) stress and oxidative stress mediated by Ras-related C3 botulinum toxin substrate 1 (RAC1) (Li et al., 2010).

## T Cell Senescence

Ageing-associated cellular senescence is also seen in the immune system, termed “immunosenescence”, and is characterised by impaired vaccination responses, a greater susceptibility to infections and the development of age-related diseases (Fulop et al., 2017). Immunosenescence effects both the innate and adaptive immune system, changes to innate immunity are thought to reflect cellular dysfunction (Shaw et al., 2013), characterised by persistent systemic inflammation in the absence of infection.

The adaptive immune system is particularly sensitive to senescence, with both humoral and T cell immunity exhibiting many phenotypic and functional hallmarks of senescence, such as short telomeres, reduced cell proliferation and increased p16 expression (Frasca et al., 2017; Callender et al., 2018). Together with a rise in the number of terminally differentiated cells secreting a highly inflammatory SASP (Frasca et al., 2017; Callender et al., 2018). However, much of the deterioration in the protective immune response has been attributed to defective T cell immunity (Nikolich-Zugich, 2005).

There is an ever-growing appreciation that senescent T cells also play a role in many chronic inflammatory and metabolic diseases (Shirakawa et al., 2016; Callender et al., 2018). Indeed, a recent paper highlighted the importance of immunosenescence in the pathogenesis of age-related disease. By selectively deleting *Ercc1*, a nuclease involved in DNA nucleotide excision repair, in haematopoietic cells, the authors created a mouse model of accelerating immune ageing. They were able to demonstrate that solid organs also showed increased senescence and damage, indicating that senescent, aged immune cells can promote systemic ageing (Yousefzadeh et al., 2021). T2D is a disease of ageing and a model of premature immunosenescence (Nikolich-Zugich, 2005). A loss of naïve CD4^+^ and CD8^+^ T cells along with a rise in late stage differentiated T cells has been reported in T2D (Giubilato et al., 2011; Lau et al., 2019). Furthermore, it has been demonstrated that the presence of senescent T cells can predict the development of hyperglycaemia in humans (Lee et al., 2019). However, both the innate and adaptive immune response in T2D show functional impairments that resemble those of ageing: poor control of infections and reduced vaccination responses together with elevated inflammatory activity (Lau et al., 2019).

Cell metabolism is a vital regulator of T cell fate and function, and the metabolic requirements of T cells change in meet the demands put upon them. However, senescent T cells exhibit mitochondrial dysfunction and consequently a reliance on glycolysis (Henson et al., 2014). Senescent T cells strongly resemble diabetic T cells in that they display impaired glucose uptake and increased fatty acid uptake but a decline in FAO (Lau et al., 2019). This abnormal glucose homeostasis observed during immunosenescence may be due to a disrupted balance between endosomal recycling and autophagy, specifically ATG9 (Henson et al., 2014). Indeed, ATG proteins have been shown to directly interact with membranes, transfer lipids between membranes and regulate lipid metabolism (Nishimura and Tooze, 2020). However, their role in altered lipid handle during T2D remains to be examined. Therefore, the alterations in the metabolism observed in senescent T cells may have the potential to drive metabolic disease.

## Senolytics and Immunomodulation

Numerous reports have demonstrated that the removal of senescent cells delays age-related tissue dysfunction, increases health span and ameliorates disease (Baker et al., 2016). Furthermore, novel drugs termed senolytics, which kill senescent cells have been shown to help maintain homeostasis in aged or damaged tissues, and postpone many age-related pathologies (Kale et al., 2020). The inflammatory secretome produced by senescent cells recruits immune cells to eliminate then but senescent cells can also interact with immune cells to avoid elimination. The expression of HLA-E by senescent fibroblasts in culture and in the skin of older humans increases and interacts with NKG2A to inhibit NK and CD8^+^ cytotoxicity (Pereira et al., 2019).

Additionally, senescent CD8^+^ T cells preferentially express more inhibitory receptors, favouring expression of the inhibitory NKG2A rather than the activatory receptor, NKG2D (Pereira et al., 2019).

Metalloproteases secreted as part of the SASP cause the shedding of MICA and MICB, ligands for the activatory receptor NKG2D, preventing NK cells and CD8^+^ T cells from interacting adding an additional barrier in the removal of senescent cells (Muñoz et al., 2019). This defective immune mediated senescent cell clearance causes senescent cells to accumulate and enhance the proinflammatory environment. Therefore, modulation of the immune system may boost the effectiveness of senolytic therapies. This could be achieved by targeting the inflammatory secretome of senescent T cells. Inhibition of p38 MAPK in senescent CD8^+^ T cells reduces the production of a T cell SASP (Callender et al., 2018) as well as increasing their proliferation, telomerase activity, and mitochondrial biogenesis (Henson et al., 2014). Blocking the inhibitory HLA-E:NKG2A signaling axis was found to improve immune clearance of senescent cells when senescent fibroblasts were incubated with NK cells and late differentiated CD8^+^ T cells (Pereira et al., 2019). Altering the suppressive activity of regulatory T cells (Tregs) may also provide a useful strategy, as both aged mice and humans have an increased proportion of Tregs which suppress T effector cell function (Song et al., 2020). A study of supercentenarians older than 110 years were found to have elevated CD4^+^ cytotoxic T cells suggestive that an increased effector function boosts longevity (Hashimoto et al., 2019). Additionally, the selectivity and efficiency of cytotoxic T cells could be reinstructed through the use of a modified T cell receptor or a chimeric antigen receptor (CAR) (June et al., 2018). The advantage of using CAR-T cells to target senescent cells is that they are able to access deep into tissues where senescent cells reside (Kale et al., 2020). Indeed, a CAR-T engineered against fibroblast activation protein found in active cardiac fibroblasts and a driver of myocardial disease, was shown to alleviate cardiac fibrosis and reverse both systolic and diastolic cardiac function (Aghajanian et al., 2019). Senescence-specific surface antigens can also serve as targets for CAR-T cells, when the urokinase-type plasminogen activator receptor, induced during senescence was engineered into a CAR-T it was found to restore tissue homeostasis in mice with liver fibrosis (Amor et al., 2020). The potential of CAR-T cell therapy has yet to be fully realised.

## Conclusion

A better understanding of immunosenescence in cardiomyopathy is needed to speed the development of novel therapeutic interventions. While several small-molecule drugs and senolytics have entered clinical trials to counter cellular senescence-associated ageing and cardiovascular disease, there remain many unanswered questions. We need a more thorough understanding of the heterogeneity of senescent cells and of the targets for potential immune cell intervention. In addition, we need to determine how immune cells interact with senescent cells in the T2D heart. Importantly, it is crucial to understand the mechanism by which senescent cells avoid immune clearance in order to develop strategies to boost the natural senolytic ability of immune cells.
